# Genetics for low correlation between Fusarium head blight disease and deoxynivalenol (DON) content in a bread wheat mapping population

**DOI:** 10.1007/s00122-019-03362-9

**Published:** 2019-05-25

**Authors:** Xinyao He, Susanne Dreisigacker, Ravi P. Singh, Pawan K. Singh

**Affiliations:** International Maize and Wheat Improvement Center (CIMMYT), Apdo. Postal 6-641, 06600 Mexico, DF Mexico

## Abstract

**Key message:**

Two QTL with major effects on DON content reduction were identified on chromosomes 3BL and 3DL, with the former showing minor and the latter showing no effects on FHB resistance.

**Abstract:**

Deoxynivalenol (DON) contamination in food and feed is a major concern regarding Fusarium head blight (FHB) infection in wheat. However, relatively less attention has been paid on DON compared to FHB. In this study, a FHB-susceptible cultivar ‘NASMA’ was hybridized with a FHB-resistant CIMMYT breeding line ‘IAS20*5/H567.71’ to generate 197 recombinant inbred lines. The population was phenotyped for FHB and associated traits including DON accumulation in spray-inoculated field experiments at CIMMYT-Mexico across four years. Genotyping was performed by using the Illumina Infinium 15 K Beadchip and SSR markers. QTL mapping results indicated that the field FHB resistance was mainly controlled by QTL at *Rht-D1* and *Vrn-A1*, along with a few minor QTL. For DON content, two major QTL were identified: the first located on chromosome 3BL (*R*^*2*^ of 16–24%), showing minor effects on FHB, and the second was on chromosome 3DL (*R*^*2*^ of 10–15%), exhibiting no effect on FHB resistance. It is likely that both DON QTL are new based on comparison with previous studies. This study indicates that resistance to DON accumulation and FHB disease could involve different genes, and the utilization of the two DON QTL in breeding could be helpful in further reducing DON contamination in food and feed.

**Electronic supplementary material:**

The online version of this article (10.1007/s00122-019-03362-9) contains supplementary material, which is available to authorized users.

## Introduction

Fusarium head blight (FHB) is a fungal disease with global importance. Prior to 1990s, it was mainly found in East Asia, Europe and the Southern Cone of South America. Since 1990s, however, its epidemics has been increasing in the USA and Canada, due to adoption of conservation agricultural practices, maize-wheat rotation and climate change (McMullen et al. 2012). In China, the disease has been spreading northward since the last two decades towards the Yellow and Huai River valleys, the main wheat production zone of China, and is becoming one of the most important wheat diseases in the region (Xu and Nicholson 2009). Unlike other wheat diseases, FHB causes not only yield loss, but also mycotoxin contamination in food and feed, severely threatening human and animal health. Worldwide, the most prevalent causal agent of FHB is *Fusarium graminearum*, which produces mainly deoxynivalenol (DON), a mycotoxin for which legislation has been made in many countries and organizations to ensure food safety (Buerstmayr et al. 2012). There has been a range of approaches developed to reduce DON contamination in food and feed; but host resistance is generally regarded as the most economical and environment-friendly method (Mesterhazy 2014; Osman et al. 2015).

Several FHB resistance mechanisms have been identified in wheat, of which type II resistance has been regarded as the most effective and stably inherited, and its measurement is straightforward with FHB severity scored after point inoculation (Bai and Shaner 2004). Type I resistance for initial infection, type III resistance for DON accumulation and type IV resistance for kernel infection are generally less investigated compared to type II resistance (Liu et al. 2009), and these components are often regarded as dependent on or highly influenced by environments and associated traits, e.g. type I resistance is frequently associated with plant height (PH) and days to heading (DH) (Mao et al. 2010; He et al. 2016a, b). Although type III resistance and type IV resistance are supposed to be independent resistance mechanism, they have shown mostly association with type I and/or type II. For example, Liu et al. (2009) summarized the QTL for different FHB resistance components and found that only 25 and 22 out of 209 QTL were for types III and IV, respectively, and none of them was independent from type I and/or type II.

Type III resistance was first reported by Miller et al. (1985), where resistant cultivars showed abilities to prevent synthesis and/or to promote degradation of DON. In their subsequent in vitro experiment, embryo callus culture of Frontana degraded 18% of ^14^C DON added to the culture, whereas that of the susceptible cultivar Casavant degraded only 5% of ^14^C DON, demonstrating the active role of type III resistance and its different expression between cultivars (Miller and Arnison 1986).

QTL exclusively associated with DON have been reported in a few studies. For example, a QTL on 2AS was found to be only responsible for DON in a Norwegian wheat cultivar NK93604, explaining phenotypic variation of 26.7%, but unfortunately data from only one environment were available (Semagn et al. 2007). In another example, QTL exclusively for DON were identified on 3A, 4B, 7A and 7B, of which only the first and the last were repeatable; and none showed phenotypic effects higher than 10% (Szabo-Hever et al. 2014), the conventional threshold for defining major QTL. Similarly, other QTL exclusively for DON were either or both of minor effects and from single environments (Szabo-Hever et al. 2014; Somers et al. 2003; Yu et al. 2008; Liu et al. 2013; Arruda et al. 2016; Lu et al. 2013).

In the present study, we report a QTL on 3DL showing consistently major effects only on DON content, and a QTL on 3BL exhibiting major effects on DON and Fusarium damaged kernels (FDK, a measurement of type IV resistance) but minor effects on field FHB resistance.

## Materials and methods

### Plant materials

A recombinant inbred line (RIL) population derived from a cross between spring wheat lines ‘NASMA’ and ‘IAS20*5/H567.71’ was used in the present study. The population was developed via the single seed decent method until F_2:7_ generation, comprising 197 individuals. The female parent NASMA with the pedigree ‘BT1149//Florence/AuroreC’ is a Moroccan variety released in 1973 (Dreisigacker et al. 2015), showing high level of FHB susceptibility. The male parent IAS20*5/H567.71 is a CIMMYT breeding line with good FHB resistance, being derived from the Brazilian variety ‘IAS20’ with a pedigree COLONIAS//FRONTANA/KENYA58.

## Field trials and phenotyping

The population was evaluated for FHB resistance in CIMMYT’s El Batan research station (altitude of 2240 metres above the sea level, coordinate 19.5°N, 98.8°W, with an average annual precipitation of 625 mm), located in the State of Mexico, Mexico. The cropping cycle is in summer from May to September when rainfall is concentrated. Field trials were conducted in 2010, 2013, 2014 and 2017 and the RILs were planted in 1-m double rows with two replications in randomized complete block design. In each plot, 10 spikes were tagged exactly at anthesis from 10 individual plants for later FHB evaluation. Inoculum comprised a mixture of five aggressive DON producing *F. graminearum* isolates following the protocols described in He et al. (2013a). At anthesis, the plots were sprayed with an inoculum of 50,000 spores/ml, and the procedure was repeated two days later to reinforce the infection. A misting system was set up in the nursery, operational from 9 am to 8 pm with 10 min of spraying each hour, to maintain a humid environment conducive for FHB infection.

Field evaluation of FHB infection was carried out at 25 days post-inoculation (dpi) on the 10 spikes marked at anthesis. Numbers of total and infected spikelets of each spike were scored for the calculation of FHB index with the formula: *FHB index* = *severity* × *incidence* (Stack and McMullen 1994), where *severity* means the averaged percentage of diseased spikelets, and *incidence* the percentage of symptomatic spikes. At full maturity, the plots were manually harvested and threshed with a belt thresher set at low wind speed in order to keep scabby kernels. FDK and DON were evaluated in 2013, 2014 and 2017. For FDK, estimation was based on a random sample in a Petri dish with a linear scale from 0 to 9, where both scabby and shrivelled kernels were regarded as FDK. For DON quantification, 20-g grain sample was ground with a coffee mill and a 2-g sub-sample was measured with the Ridascreen Fast DON ELISA kit (RBiopharm GmbH, Darmstadt, Germany) following the manufacturer’s instructions. PH and DH were recorded in all the experiments except for PH in 2010.

In order to exclude the possibility that a DON QTL is associated with grain filling period (GFP) or grain filling rate (GFR), an additionally experiment was performed in glasshouse in spring 2018, where DH, days to maturity (DM) and thousand kernel weight (TKW) were taken. GFP was estimated via subtracting DH from DM, and GFR via dividing TKW with GFP.

## Statistical analyses

Analysis of variance (ANOVA) was carried out with the PROC GLM in SAS program ver. 9.2, and Pearson correlation coefficients were calculated using the PROC CORR function. The ANOVA output was used for calculating the heritability estimates, using the formula $H^{2} = \sigma_{g}^{2} /(\sigma_{g}^{2} +  \sigma_{g*y}^{2} /y +  \sigma_{e}^{2} /ry)$, in which $\sigma_{g}^{2}$ stands for genetic variance, $\sigma_{g*y}^{2}$ for genotype-by-year interaction, $\sigma_{e}^{2}$ for error variance, *y* for the number of years, and *r* for the number of replications (Lu et al. 2013).

## Genotyping

The population had previously been genotyped with a set of SSR and KASP markers in Dreisigacker et al. (2015) for mapping Septoria tritici blotch resistance. In the present study, more markers were applied, including the Illumina Infinium 15 K Beadchip provided by TraitGenetics GmbH, Germany. Markers with missing data points greater than 20% and segregation ratio beyond the range 0.5–2.0 were not used in subsequent analysis.

## Linkage and QTL mapping

Linkage mapping was performed with the JoinMap v.4 software (Van Ooijen 2006), where groupings were based on LOD scores from 5 to 10, and ordering within each linkage group (LG) was calculated with the maximum likelihood algorithm. LGs were assigned to chromosomes according to the Illumina 90 K SNP map in Wang et al. (2014). QTL mapping was performed with MapQTL v6.0 (Van Ooijen 2009), in which interval mapping (IM) was first completed to detect potential QTL for each trait, followed by multiple QTL mapping (MQM) for each QTL, using the tightly linked markers to each QTL detected in IM as cofactors. QTL were defined as significant and were reported if they were over the LOD threshold of 3.0 in at least one environment or over the threshold of 2.0 in multiple environments. In order to eliminate the confounding effects of PH and DH on FHB parameters, MQM was also performed with PH and DH as covariates, to determine whether a QTL represents real resistance gene (He et al. 2016a). LGs and LOD curves were drawn with the software MapChart ver. 2.3 (Voorrips 2002).

## KASP assays for SNP markers associated with DON

SNP markers associated with major QTL for DON were transformed into KASP assays to facilitate marker-assisted selection (MAS). The RIL population was genotyped with the KASP, and the markers were re-mapped. PCR were carried out in a system of 4 µl containing 2 × KASPV4.0 Mastermix, 120 nM of each allele-specific primers and 300 nM of common primer. Cycling condition comprised 15 min at 94 °C, 20 cycles of 10 s at 94 °C, 5 s at 57 °C, and 10 s at 72 °C. PCR products were analysed with a PHERAstar FS microplate reader (BMG Labtech, Ortenberg, Germany).

## Database exploration

The T3/Wheat website (https://triticeaetoolbox.org/wheat/) was used for extracting genotypic data of Frontana for markers in the 3BL and 3DL QTL regions. The JBrowse tool in T3/Wheat was used to retrieve annotated genes in the QTL regions. Physical positions of markers in IWGSC RefSeq v1.0 were obtained from either T3/Wheat or URGI (https://urgi.versailles.inra.fr/blast_iwgsc/blast.php) via BLAST searches.

## Results

### Phenotypic analysis

**Fig. 1:**
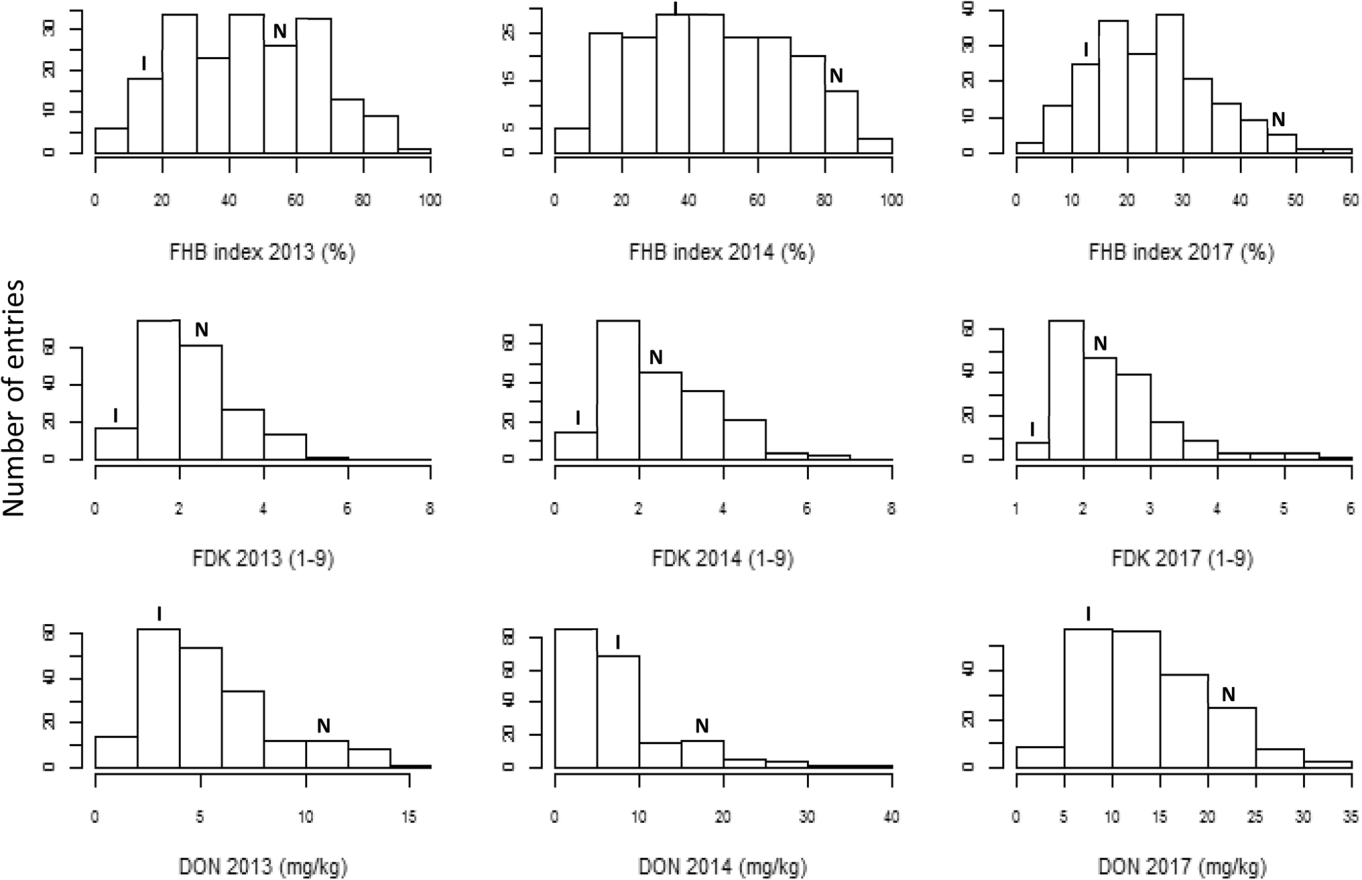
Histograms for Fusarium head blight (FHB), Fusarium damaged kernels (FDK), and deoxynivalenol (DON) content. Phenotypic ranges of the two parents are indicated, where *I* stands for IAS20*5/H567.71 and *N* for NASMA. FHB chart for 2010 was not drawn due to the layout limit

FHB disease pressure varied greatly among years, of which 2010 witnessed the lowest FHB development with a grand mean FHB index of 17.5%, followed by 2017 (24.0%), 2013 (45.1%) and 2014 (46.5%). Distribution of lines for FHB resistance was continuous and transgression at both directions could be observed, whereas that for FDK and DON was also continuous but skewed towards the resistance side (Fig. 1). ANOVA demonstrated that ‘year’ was the major source of variation for both FHB and DON; nevertheless, significant effects of ‘genotype’ were found for all three FHB related parameters, as well as that of ‘genotype × year’ for FHB and DON (Table 1). High heritability estimates from 0.79 to 0.84 were achieved for the three traits (Table 1).

**Table 1:** Analysis of variance for Fusarium head blight (FHB), Fusarium damaged kernels (FDK), deoxynivalenol (DON) content and their heritability estimates

Traits	Source	DF	Mean square	*F* value	*P* value	Heritability
FHB	Genotype	196	1281.93	15.13	< 0.0001	0.84
	Year	3	65,477.30	772.68	< 0.0001	
	Genotype × year	583	221.79	2.62	< 0.0001	
	Rep. (Year)	3	1349.74	15.93	< 0.0001	
	Error	584	84.74			
FDK	Genotype	196	5.36	5.00	< 0.0001	0.79
	Year	2	7.83	7.31	0.0007	
	Genotype × year	289	1.12	1.04	0.3273	
	Rep. (year)	3	60.74	56.71	< 0.0001	
	Error	567	1.07			
DON	Genotype	196	132.21	8.03	< 0.0001	0.81
	Year	2	7015.40	426.34	< 0.0001	
	Genotype × year	391	25.34	1.54	< 0.0001	
	Rep. (year)	3	209.67	12.74	< 0.0001	
	Error	585	16.46			

Positive correlation was found among all FHB parameters, but some of them were non-significant or low, especially the correlation between FHB and DON (Table 2). PH and DH showed mostly negative correlation with FHB traits, of which PH showed more stable correlation with FHB, whereas DH exhibited variable correlation, from non-significant to highly significant; however, they showed low and usually non-significant correlation with FDK and DON (Table 3). Regarding the two traits related to grain filling, GFP did not show significant correlation with FHB traits, whereas GFR showed only marginally negative correlation with FHB index, but not with FDK nor DON (Table 3).

**Table 2:** Pearson correlation coefficients among Fusarium head blight (FHB), Fusarium damaged kernels (FDK) and deoxynivalenol (DON) content

	FHB10	FHB13	FHB14	FHB17	FDK13	FDK14	FDK17	DON13	DON14
FHB13	0.63**								
FHB14	0.66**	0.85**							
FHB17	0.52**	0.52**	0.53**						
FDK13	0.42**	0.30**	0.31**	0.22*					
FDK14	0.42**	0.20*	0.28**	0.32**	0.61**				
FDK17	0.42**	0.37**	0.40**	0.29**	0.59**	0.56**			
DON13	0.20*	0.31**	0.33**	0.15	0.36**	0.24*	0.38**		
DON14	0.21*	0.23*	0.29**	0.12	0.34**	0.35**	0.41**	0.71**	
DON17	0.23*	0.31**	0.07	0.16	0.32**	0.36**	0.47**	0.66**	0.66**

Numbers behind the trait names indicate the years in which the traits were measured, i.e. ‘13’ for the year 2013, ‘14’ for 2014 and ‘17’ for 2017

^*^P < 0.01; **P < 0.0001

**Table 3:** Pearson correlation coefficients between FHB parameters (Fusarium head blight (FHB), Fusarium damaged kernels (FDK), deoxynivalenol (DON) content) and additionally investigated traits

	Days to heading	Plant height	Grain filling period	Grain filling rate
FHB10	− 0.26**	− 0.44**	0.06	− 0.24*
FHB13	− 0.49**	− 0.56**	0.13	− 0.23*
FHB14	− 0.41**	− 0.45**	0.15	− 0.24*
FHB17	− 0.01	− 0.32**	0.05	− 0.26**
FDK13	− 0.11	− 0.22*	0.00	− 0.17
FDK14	0.11	− 0.19*	− 0.04	− 0.10
FDK17	− 0.27**	− 0.31**	− 0.01	− 0.14
DON13	− 0.21*	− 0.23*	− 0.11	− 0.01
DON14	− 0.16	− 0.01	− 0.05	0.08
DON17	− 0.23*	− 0.19*	− 0.13	0.02

Numbers behind the trait names indicate the years in which the traits were measured, i.e. ‘10’ for the year 2010, ‘13’ for 2013, ‘14’ for 2014 and ‘17’ for 2017. Days to heading and plant height were measured in the same field trials as FHB parameters, whereas grain filling period and grain filling rate were evaluated in glasshouse in 2018

^*^P < 0.01; **P < 0.0001

## Genotyping and linkage analysis

Totally 1281 non-redundant markers of high quality were used for constructing LGs, including 80 SSRs and functional markers for genes *Rht-B1*, *Rht-D1*, *Vrn-A1* and *Tsn1*. Thirty one LGs were generated, representing all 21 wheat chromosomes and covering 3163 cM, with an average genetic distance of 2.5 cM between markers. D genome LGs usually had poor marker coverage and often fragmented, especially LGs for 4D (3 markers, 20 cM), 6D (4 LGs, 21 markers, 38 cM) and 7D (3 LGs, 19 markers, 46 cM), whereas all other LGs were of genetic lengths > 100 cM.

## QTL mapping

**Fig. 2:**
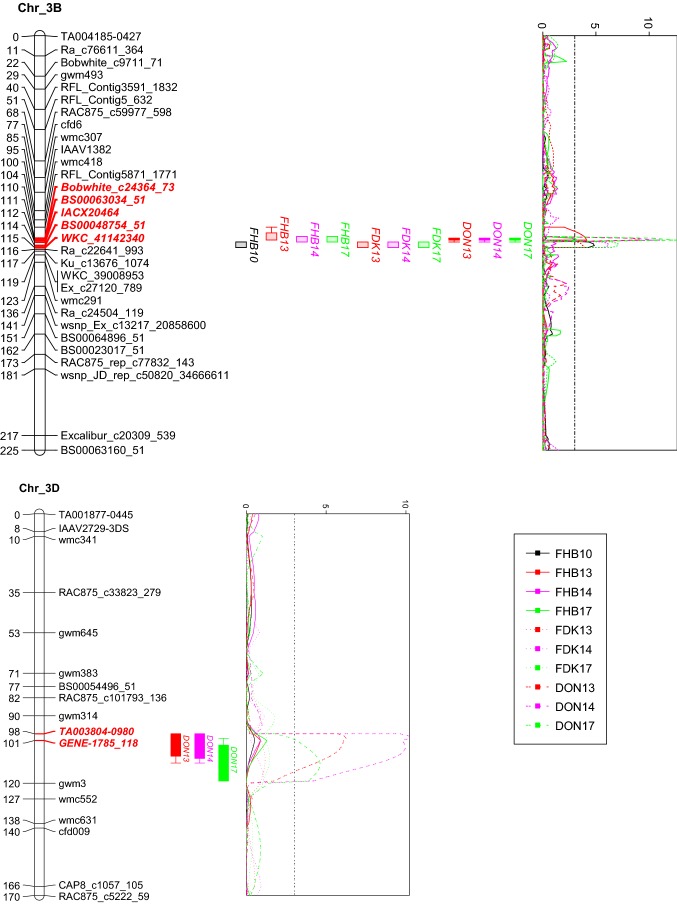
QTL profiles for Fusarium head blight (FHB), Fusarium damaged kernels (FDK), and deoxynivalenol (DON) content on chromosomes 3BL and 3DL across environments. Genetic distances are shown in centimorgans to the left of the chromosomes. A threshold of 3.0 is indicated by a dashed vertical line in the LOD graphs. Only framework markers are presented except for the QTL regions, where the markers are bolded and highlighted in red (color figure online)

Due to the wide segregation of both PH and DH, their underlying genes *Rht-D1*, *Rht-B1*, *Vrn-A1*, and a QTL on 5BL for DH (close to *Vrn-B1* according to the IWGSC RefSeq v1.0) showed significant effects on FHB index, but except *Rht-D1* that had minor effects on FDK, none of them showed effects on FDK or DON (Table 4). The only FHB QTL that was independent from PH and DH was the one on chromosome 3BL, exhibiting minor but stable effects of 4.4–8.7%. QTL for FDK were mostly minor and unstable; except the 3BL QTL mentioned above as a minor QTL for FHB, which demonstrated higher effects on FDK, ranging from 7.5 to 13.6%. Interestingly, the same 3BL QTL showed even higher effects for DON, with phenotypic variation explained from 15.8 to 23.9% (Table 4, Fig. 2). QTL intervals for DON and FDK on 3BL are different, overlapping at the marker IACX20464, whereas those for FHB had even wider ranges (Fig. S1). The other major QTL for DON was localized on chromosome 3DL, having phenotypic effects of 10.3–15.3% while exhibiting no effect on neither FHB nor FDK (Table 4, Fig. 2). When DH and PH were used as covariates, all phenology-associated QTL on 4BS (*Rht-B1*), 4DS (*Rht-D1*), 5AL (*Vrn-A1*) and 5BL (*Vrn-B1*) became non-significant for FHB, together with a minor QTL on 2DL, whereas a few minor QTL on 2BL, 3BS, 4BL and 5AS became significant for FHB or FDK (Table S1). The two major QTL on 3BL and 3DL, as well as minor QTL on 1BL, 2AL and 7AC (at the centromere region of 7A) remained significant when covariates were used (Table S1).

**Table 4:** Phenotypic effects of QTL for Fusarium head blight (FHB), Fusarium damaged kernels (FDK), and deoxynivalenol (DON) content

Chr	Position (cM)	Left marker	Right marker	FHB index				FDK			DON content			*R* source	Traits associated
				2010	2013	2014	2017	2013	2014	2017	2013	2014	2017		
1BL	66.3–71.6	wmc419	BS00064032_51									**4.4**		I	
2AL	86.9–98.7	WEC_4379619	BS00039406_51					**7.4**						I	
2DL	15.7–18.9	tplb0021c10_951	GENE-0638_732								**5.4**			N	
3BL	109.6–115.5	BW_c24364_73	wKc31407_41142340	**8.7**	**4.8**	**4.4**	**6.6**	**7.5**	**12.3**	**13.6**	**15.8**	**20.0**	**23.9**	I	
3DL	89.4–119.7	TA003804-0980	gwm3								**10.5**	**15.3**	**10.3**	I	
4BS	44.1–46.3	Rht-B1	BS00021984_51		**5.5**									N	PH
4DS	0–10.7	Rht-D1	barc105	**9.5**	**12.7**	**9.2**	**7.7**	4.9		3.9				I	PH
5AL	147.6–147.9	WEC_5013188	Vrn-A1	3.9	**21.2**	**21.7**								I	DH, PH
5BL	92.1–92.4	BS00022673_51	R_c539_1789		**4.8**	3.4	3.9							N	DH
7AC	115.6–119.8	WERC_70021470	Ku_c97425_164						**7.5**					N	
Accumulated percentage of variation explained				22.1	49.0	38.7	20.0	19.8	19.8	22.9	26.3	39.7	34.2		

The percentage of phenotypic variation explained in the multiple regression models is shown. QTL are listed if they were over the LOD threshold of 3 (in bold) in at least one environment

*I* IAS20*5/H567.71, *N* NASMA, *PH* plant height, *DH* days to heading

**Fig. 3:**
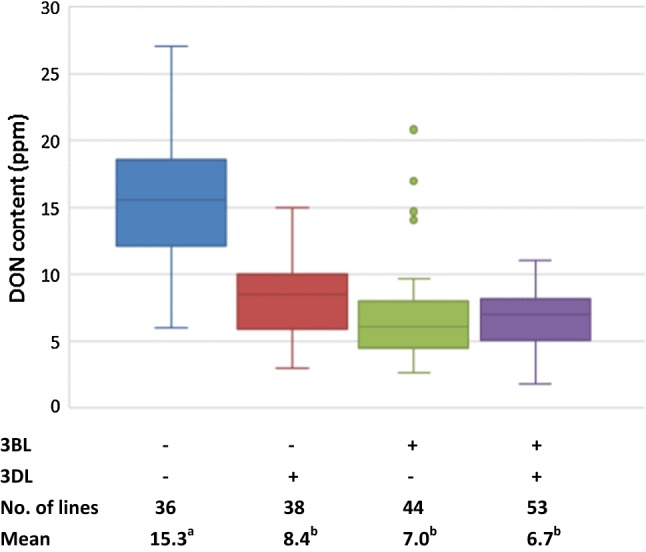
Phenotypic effects of different allele combinations for the 3BL and 3DL QTL for DON. Different letters following mean values indicate significant difference at alpha = 0.01

Resistance alleles at both 3BL and 3DL QTL were contributed by the resistant parent IAS20*5/H567.71. The two QTL showed only partial additive effects on DON, i.e. resistance alleles from either or both QTL conferred significant reduction of DON, but no significant difference was detected among their combinations (Fig. 3). Nevertheless, stacking both QTL could be beneficial, considering that the corresponding group had the lowest mean value, narrow DON range and no outlier (Fig. 3).

QTL for GFP were found only on 6AS and 7AS, and those for GFR were on 4BS (*Rht-B1*), 4DS (*Rht-D1*), 6BS and 6BL (Table S2). Apart from QTL at *Rht-B1* and *Rht-D1* that showed association with FHB index, none of these QTL was associated with FHB related traits.

DH was mainly controlled by *Vrn-A1*, explaining phenotypic variation from 28.5 to 42.7%. *Vrn-B1* on 5BL showed minor yet consistent effects and a QTL on 7AS was significant only in 2013 and 2017 (Table S3). As for PH, both *Rht-B1* and *Rht-D1* were segregating, exhibiting phenotypic effects of roughly 30 to 40%. Additionally, *Vrn-A1* were significant for PH in two years, with minor effects of around 3% (Table S3).

## KASP assays for 3BL and 3DL QTL and database mining

Four SNPs from each 3BL and 3DL QTL regions were tested with KASP assays, of which one for 3BL and two for 3DL turned out to be successful (Table 5, Table S4). These three KASPs were used for genotyping the population, and the results showed that the two KASPs on chromosome 3DL co-segregated with the corresponding SNPs of the Illumina Infinium 15 K Beadchip, whereas the KASP on chromosome 3BL exhibited a tight linkage with RAC875_c24504_119, with a genetic distance of 0.5 cM (data not shown).

**Table 5:** Haplotyping results of KASPs for the 3BL and 3DL QTL on parents of the ‘NASMA’ × ‘IAS20*5/H567.71’ population and ancestors of the resistant parent ‘IAS20*5/H567.71’

Marker name	KASP ID	SNP	Chr	Resistant allele	NASMA	IAS20*5/H567.71	IAS20	Kenya58	Colonias	Frontana
GENE-1785_118	IWB32653	A/G	3D	G	A	G	G	G	A	A
TA003804-0980	IWB65745	C/T	3D	C	T	C	C	C	C	C
RAC875_c24504_119	IWB55664	C/T	3B	C	T	C	C	C:T	T	C

These three KASPs were used to investigate the source of resistance alleles via genotyping parents of the population and ancestors of the resistant parent. As expected, the male parent IAS20*5/H567.71 harboured resistance alleles at all three loci, as well as its parent IAS20 (Table 5). Then, the three parents of IAS20 (Kenya58, Colonias and Frontana) were investigated, and the results indicated that resistance source of the 3BL QTL might be Frontana, whereas that of the 3DL QTL might be Kenya58 (Table 5).

Considering the large distance between the KASP on 3BL and the QTL peak region (Fig. 2), haplotype data for Frontana were retrieved from the T3/Wheat website. The two SNPs that are in the core QTL region of DON, Bobwhite_c24364_73 and BS00063034_51, showed resistant and susceptible genotypes, respectively, making it inconclusive regarding whether Frontana has the 3BL QTL or not (Table S5, Fig. S1). On the other hand, the susceptible genotypes at two additional loci in the 3DL region, D_GA8KES402JVT1Y_74 and BS00067163_51, provide further evidence for that Frontana is unlikely the resistance source for the 3DL QTL (Table S5, Fig. S1).

In order to saturate the 3DL region to find more closely linked markers, 39 additional SNPs in the Illumina 90 K panel between TA003804-0980 and *gwm3* (inferred based on their physical locations in the IWGSC RefSeq v.1.0) were transformed into KASPs for genotyping the population, but none of them showed polymorphism (Fig. S2). Based on QTL profiles for the 3DL QTL, the underlying gene is most likely located in the vicinity of three co-segregating SNPs, GENE-1785_118, BS00067163_51 and D_GA8KES402JVT1Y_74 (Fig. S1). The 1.2-Mb chromosome region harbouring the three SNPs contains 31 high-confidence genes (Table S6), in which a candidate gene might be, but it is also likely that Chinese Spring does not have the underlying gene, considering that two of the three SNPs mentioned above had no BLAST hit in Chinese Spring genome (Fig. S2).

The QTL region for DON on 3BL extends from physical position 672,961,307 to 722,359,301 when using Bobwhite_c24364_73 and IACX20464 as flanking markers, harbouring 385 high-confidence genes (data not shown). Fine mapping is needed to narrow down the QTL region for subsequent work on the identification and characterization of the underlying gene.

## Discussion

The public concern on FHB is more on its metabolic products, especially DON; however, due to technical and cost reasons, many studies on FHB did not measure DON, simply assuming varieties with low FHB have low DON, and vice versa, which, however, is not always true. For example, north-western China is of dry climate and FHB epidemic has been rare, but a recent survey indicated that 82.9% of wheat samples harvested in the region were contaminated with DON at an average concentration of 0.5 mg kg^−1^, and 10% of the contaminated samples showed DON content higher than the Chinese threshold for DON (1.0 mg kg^−1^) (Zhao et al. 2018b). In Canada, DON has been given higher weight than FHB incidence and severity since the last few years. An incidence/severity/DON (ISD) index is used in Canada to evaluate the FHB resistance of a cultivar, which is calculated with ISD = (0.3 incidence) + (0.3 severity) + (0.4 DON) as described in the Prairie Recommending Committee for Wheat, Rye and Triticale operating procedures (Ver. 27 Nov 2013, Updated 5 December 2015, available at https://www.pgdc.ca/committees_wrt.html). Recently, the formula has been modified to give even higher weight to DON, i.e. ISD = (0.2 incidence) + (0.2 severity) + (0.6 DON), and a cultivar ‘Zealand’ was released due to its good DON resistance, albeit its FHB resistance was rated as from intermediate to susceptible in different environments (Spaner et al. 2018).

FHB infection is a prerequisite of DON contamination, which makes people to expect high correlation between the two traits. However, it must be pointed out that two conditions must be met so that high correlation be observed, i.e. the absence of both late infection and active type III resistance; otherwise, very low or even negative correlation could be found between FHB and DON, as reviewed in Paul et al. (2005). Late infection is outside the range of the current study, but evidence has shown that it usually does not cause obvious FHB symptom, whereas leads to high DON accumulation (Del Ponte et al. 2007). As for type III resistance, the mechanism was initially proposed by Miller et al. (1985) and validated for the first time by Miller and Arnison (1986), in which Frontana showed an in vitro ability of detoxification. Based on this work, the term ‘type III resistance’ was proposed in Wang and Miller (1988) to represent DON detoxification or prevention in wheat under FHB infection. However, this type of resistance has received less attention compared to type I resistance and II resistance and the underlying genes are largely unknown, except for the UDP glycosytransferase (UGT) gene family, which are known as detoxification agents that transform DON into DON-3-glucoside (D3G) that is less toxic to plants (Lemmens et al. 2016). But so far no UGT gene has turned out to be responsible for known FHB/DON resistance QTL with major effects (Zhao et al. 2018a).

In the current study, artificial inoculation was performed at anthesis and the natural FHB infection rate in the experimental station is low (He et al. 2013a), minimizing the effects of late infection, and thus, the low correlation between FHB and DON must be ascribed to the presence of active type III resistance, just like the similar findings in oats (He et al. 2013b). Our QTL mapping results indicated the presence of major DON QTL on 3BL and 3DL, with the former showing minor effects on FHB and the latter exhibiting effects exclusively on DON. On the centromere region of chromosome 3B, many QTL have been reported with the chromosome designation of 3BSc or 3BL (Liu et al. 2013; Somers et al. 2003), conferring resistance mostly to FHB, but also to DON in Wangshuibai (Yu et al. 2008), Ernie (Liu et al. 2013) and Truman (Islam et al. 2016). When aligned to chromosome 3B of the IWGSC RefSeq v1.0, QTL range of the current study overlapped only with the QTL observed in Arina (Buerstmayr and Buerstmayr 2015) and Wangshuibai (Yu et al. 2008) (Fig. S3). For Arina, Paillard et al. (2004) also reported a QTL on 3BL, being located within the range of that reported by Buerstmayr and Buerstmayr (2015) and not overlapped with our 3BL QTL, implying that the 3BL QTL in Arina is unlikely the same as in IAS20. As for the 3BL QTL in Wangshuibai, its peak position is at *gwm376* near the centromere (Yu et al. 2008), whereas ours is located far from the centromere (Fig. S3). Thus, it is very likely that the 3BL reported in the current study is new. Similar approach was utilized to compare the positions of FHB related QTL on 3DL, and the one reported in the present study does not overlap with any of the previously reported QTL (Fig. S3), and considering its unique feature conferring resistance exclusively to DON, it must be a new QTL.

DON serves as a virulence factor and mostly associated with type II resistance (Bai et al. 2002), and thus, it was unexpected to find QTL associated primarily or exclusively with DON. In order to exclude the possibility that they are actually grain filling related genes having confounding effects on DON (Bai et al. 2018), additional experiment was performed in glasshouse to map grain filling related traits in this population. Mapping results did not indicate any effect of the 3BL and 3DL chromosome regions on GFP or GFR, and thus, the two QTL must not be false-positive. The marginal negative correlation between GFR and FHB could be due to the pleiotropic effects of dwarfing genes *Rht-B1* and *Rht-D1*, which showed significant effects on both GFR and FHB, and since the two genes exhibited very weak and no effect on FDK and DON, respectively, no such correlation could be found between GFR and FDK/DON. As for the reason why these two QTL showed weak or no effect on FHB, it could be ascribed to the late expression of the two underlying genes. The gene for 3BL might be expressed in spike tissues in late grain development stages, starting before 25 dpi, because its effects on FHB that was evaluated at 25 dpi was still big enough to be detected. On the other hand, the gene for 3DL must be expressed very late in spike, or exclusively in grain, making its effects being non-significant not only for FHB, but also for FDK.

The ability of Frontana to tolerate or degrade DON has been reported in several studies (Miller and Arnison 1986; Miller and Ewen 1997; Wang and Miller 1988), but most QTL mapping studies on Frontana or its derivatives did not measure DON, except for Szabo-Hever et al. (2014) reporting 12 Frontana-derived QTL for DON resistance, exhibiting a quantitative control of this trait in Frontana. It is noteworthy that no QTL on 3BL nor 3DL was reported in Szabo-Hever et al. (2014). In our results, Frontana appeared not having the 3DL QTL, whereas it is still inconclusive regarding whether it has the 3BL QTL. Of the three ancestors of IAS20, Frontana is the only one that have shown FHB resistance in multiple studies, so it is still possible that Frontana has the QTL.

It was unexpected that Kenya58 may have the 3DL QTL. This is an old cultivar released in 1937 in Kenya, with a pedigree of RED-EGYPTIAN/KENYA-BF-4–3-B-10-V-1 (https://wheatpedigree.net/sort/show/31523). Although no FHB data on this cultivar are available, as far as we know, it may not have good FHB resistance, considering the low FHB disease pressure in Kenya. However, by saying this, we are ignoring its potential in DON detoxification/prevention. FHB and DON are two closely related yet different traits; however, far more efforts have been put on the former in the last two decades, assuming good FHB resistance leads to good DON resistance (Liu et al. 2009). However, it should be noted that the valuable trait ‘DON detoxification/prevention’ could be masked by high level of FHB resistance, which inhibits fungal colonization to a large extend, giving little chance for DON detoxification/prevention to express. In practice, few cultivar has high level of FHB resistance, enabling the *Fusarium* pathogen to colonize spike tissues, and thus, DON detoxification/prevention becomes very important in terms of food safety (Bai and Shaner 2004). The results of this study imply that DON detoxification/prevention may be a largely unexplored resource in FHB resistance breeding, and the related genes may present in many moderately resistant or even susceptible varieties.

Nevertheless, it should be noted that QTL conferring strong DON resistance but weak or no FHB resistance, like the ones on 3BL and 3DL in this study, should be utilized together with FHB resistance genes like *Fhb1*, *Fhb2,* etc., to achieve a holistic resistance, leading to high resistance to both FHB and DON. Based on haplotyping results of 1,300 CIMMYT breeding lines using KASPs for the 3BL and 3DL QTL, frequency of resistant alleles at the two QTL was very low in CIMMYT germplasm (might not exceed 5%, data not shown), implying significant breeding efforts in increasing their frequency in CIMMYT gene pool are needed.

Our results emphasized the importance of measuring DON in FHB resistance breeding programs. However, the capacity of testing DON may not be available for some breeding programs, and in that case, FDK is strongly recommended in addition to FHB, because FDK usually shows better correlation with DON than FHB, as shown in the present study, as well as in previous studies (Mesterhazy et al. 2005; Lu et al. 2013; Paul et al. 2005).

### Author contribution statement

PS and XH conceived and designed the experiments; XH and PS performed field trials; SD conducted all genotyping activities; RS provided plant materials; XH analysed the data and wrote the first draft of the manuscript, and all co-authors contributed and approved the final draft of the manuscript.

## Electronic supplementary material

Below is the link to the electronic supplementary material.

Supplementary file1 (DOCX 96 kb)
