# Supplementary material for: Genetics for low correlation between Fusarium head blight disease and deoxynivalenol (DON) content in a bread wheat mapping population
Source: Theor Appl Genet. 2019 May 25;132(8):2401–11. doi: 10.1007/s00122-019-03362-9 (PMC6647199; doi:10.1007/s00122-019-03362-9)
Supplement: Supplementary file 1 — Supplementary file1 (DOCX 96 kb) [file 122_2019_3362_MOESM1_ESM.docx]

**Electronic Supplementary Materials**

**Article title:** “Genetics for low correlation between Fusarium head blight and deoxynivalenol (DON) content in a bread wheat population”

**Journal:** Theoretical and Applied Genetics

**Authors:** Xinyao He^1^, Susanne Dreisigacker^1^, Ravi P. Singh^1^, Pawan K. Singh^1*^

^1^ International Maize and Wheat Improvement Center (CIMMYT), Apdo. Postal 6-641, 06600 Mexico DF, Mexico

**Name, affiliation, and email of corresponding author:**

Pawan K. Singh

Global Wheat Program, International Maize and Wheat Improvement Center (CIMMYT)

[pk.singh@cgiar.org](mailto:pk.singh@cgiar.org)

**Table S1** Phenotypic effects of QTL for Fusarium head blight (FHB), Fusarium damaged kernels (FDK), and deoxynivalenol (DON) content, using days to heading and plant height as covariates

| Chrom. | Position | Left marker | Right marker | FHB index | | | | FDK | | | DON content | | | R source |
| --- | --- | --- | --- | --- | --- | --- | --- | --- | --- | --- | --- | --- | --- | --- |
|  |  |  |  | 2010 | 2013 | 2014 | 2017 | 2013 | 2014 | 2017 | 2013 | 2014 | 2017 |  |
| 1BL | 66.3-71.6 | wmc419 | BS00064032_51 |  |  |  |  |  |  |  |  | **4.4** |  | I |
| 2AL | 86.9-98.7 | WEC_4379619 | BS00039406_51 |  |  |  |  | **8.4** | 4 |  |  |  |  | I |
| 2BL^#^ | 109.1-112.6 | BW_c27816_523 | Ra_c15365_530 |  |  |  | **9.2** |  |  |  |  |  |  | I |
| 3BS^#^ | 11.4-14.4 | BS00079989_51 | tplb0059m03_1516 |  |  |  | **5.9** |  |  |  |  |  |  | N |
| 3BL | 109.6-126.6 | BW_c24364_73 | WEC_58898157 | **7.8** | **4.5** | **6.6** | **5.6** | **5.8** | **11.9** | **11.9** | **17.3** | **17.3** | **22.6** | I |
| 3DL | 89.4-119.7 | TA003804-0980 | gwm3 |  |  |  |  |  |  |  | **11.6** | **13.4** | **8.8** | I |
| 4BL^#^ | 82.6-83.3 | Ra_c107130_384 | IAAV8848 |  |  |  | **4.9** |  |  |  |  |  |  | N |
| 5AS^#^ | 0.9-1.8 | WEC_1748920 | WEC_6612231 |  |  |  |  | **4.9** |  |  |  |  |  | I |
| 7AC | 112.6-121.3 | gwm260 | WKC_60941831 | 3.3 |  |  |  | 3.3 | 4.2 |  |  |  |  | N |
| Accumulated percentage of variation explained | | | | 11.1 | 4.5 | 6.6 | 25.6 | 22.4 | 20.1 | 11.9 | 28.9 | 35.1 | 31.4 |  |

The percentage of phenotypic variation explained in the multiple regression models is shown

QTL are listed if they were over the LOD threshold of 3 (in bold) in at least one environment

# denotes QTL detectable only when covariates were used

*I* IAS20*5/H567.71, *N* Nasma

**Table S2** QTL for grain filling period (GFP) and grain filling rate (GFR)

| Trait | Chrom. | Position | Left marker | Right marker | Phenotypic effects | Source |
| --- | --- | --- | --- | --- | --- | --- |
| GFP | 6AS | 56.6-64.6 | T_c10412_650 | BW_c19647_159 | 4 | N |
|  | 7AS | 66.8-69.1 | Ex_c34807_431 | Ra_c9427_300 | **9.1** | N |
|  | Accumulated percentage of variation explained | | | | 13.1 |  |
| GFR | 4BS | 44.1-46.3 | Rht-B1 | BS00021984_51 | **10.6** | N |
|  | 4DS | 0.0-10.7 | Rht-D1 | barc105 | **5.8** | I |
|  | 6BS | 19.4-24.7 | TA004132-0670 | Ra_c26860_648 | **6.0** | I |
|  | 6BL | 70.4-70.7 | IACX5699 | Ku_c25725_1921 | **6.4** | N |
|  | Accumulated percentage of variation explained | | | | 28.8 |  |

The percentage of explained phenotypic variation in 2018 is shown. QTL with LOD threshold over 2 are listed and those with LOD over 3 are *bolded*. Source of allele for high phenotypic value is indicated. *I* IAS20*5/H567.71, *N* Nasma

**Table S3** QTL for days to heading (DH) and plant height (PH)

| Trait | Chrom. | Position | Left marker | Right marker | Phenotypic variation explained | | | | Source of high DH or PH values |
| --- | --- | --- | --- | --- | --- | --- | --- | --- | --- |
|  |  |  |  |  | 2010 | 2014 | 2015 | 2017 |  |
| DH | 5AL | 147.6-147.9 | WEC_5013188 | Vrn-A1 | **34.3** | **30.6** | **42.7** | **28.5** | N |
|  | 5BL | 81.4-91.3 | IAAV5992 | BS00065128_51 | **9.1** | **7.1** | 4.4 | **6.3** | I |
|  | 7AS | 66.8-68.8 | Ex_c34807_431 | BS00063555_51 |  | **7.3** |  | 3.4 | N |
|  | Accumulated percentage of variation explained | | | | 43.4 | 45 | 47.1 | 38.2 |  |
| PH | 4BS | 44.1-46.3 | Rht-B1 | BS00021984_51 | NA | **38** | **43.8** | **33.8** | N |
|  | 4DS | 0-10.7 | Rht-D1 | barc105 | NA | **38.8** | **41.2** | **31.8** | I |
|  | 5AL | 147.6-147.9 | WEC_5013188 | Vrn-A1 | NA | 3.1 | 2.7 |  | I |
|  | Accumulated percentage of variation explained | | | | NA | 79.9 | 87.7 | 65.6 |  |

The percentage of explained phenotypic variation is shown. QTL with LOD threshold over 2 are listed and those with LOD over 3 are *bolded*. Data for PH was not available for 2010. Source of allele for high phenotypic value is indicated. *I* IAS20*5/H567.71, *N* Nasma

**Table S4** KASP assay information for SNPs linked to QTL on chromosomes 3BL and 3DL

| Marker name | KASP ID | SNP | Chr | Resistant allele | Primer A | Primer B | Common primer |
| --- | --- | --- | --- | --- | --- | --- | --- |
| GENE-1785_118 | IWB32653 | A/G | 3D | G | gcatgctaactaatggGcacA | gcatgctaactaatggGcacG | ggtggccaaacacaaacaga |
| TA003804-0980 | IWB65745 | C/T | 3D | C | tcacgcgatccgagttactaaC | tcacgcgatccgagttactaaT | gtggcagatgagggacgata |
| RAC875_c24504_119 | IWB55664 | C/T | 3B | C | taggcagcccgtatccAgC | taggcagcccgtatccAgT | aactttaaggggattgtgaggat |

**Table S5** Haplotype prediction of Frontana at 3BL and 3DL QTL, using data available from the T3/Wheat website

|  | SNP | Genetic position | S allele | R allele | SNP call in Frontana | TCAP Code | Physical position on the target region in RefSeq v1.0 | Possible hits on other chromosomes^#^ |
| --- | --- | --- | --- | --- | --- | --- | --- | --- |
| 3BL | RFL_Contig5871_1771 | 103.997 | T | C | C | IWB65143 | 664896524 | None |
|  | BW_c24364_73 | 109.633 | G | T | T | IWB1821 | 672961307 | None |
|  | BS00063034_51 | 111.386 | T | C | T | IWB8984 | 685637669 | None |
|  | IACX20464 | 112.293 | G | A | G | IWB35831 | 722359499 | 3D |
|  | BS00048754_51 | 113.867 | A | G | NA | IWB8515 | 682905538 | None |
|  | wKu_c31407_41142340 | 115.47 | C | T | NA | IWA6900 | 708953422 | None |
| 3DL | TA003804-0980* | 98.107 | T | C | C | IWB65745 | 562701194 | 3A |
|  | GENE-1785_118* | 101.067 | A | G | A | IWB32653 | No hit | 3A |
|  | BS00067163_51 | 101.067 | A | G | A | IWB9976 | No hit | 3A |
|  | D_GA8KES402JVT1Y_74 | 101.067 | G | A | G | IWB17930 | 571550398 | None |

^*^ Results for the two markers were obtained from the KASP assays in this study, rather than predicted from T3/Wheat

^#^ Results are listed only when blast scores on alternative loci are comparable to those on target loci

**Table S6** High confidence gene prediction in the 3DL QTL region in Chinese Spring (the IWGSC RefSeq v1.0)

| **Gene-ID** | **chr** | **start/bp** | **end/bp** | **Blast-Hit-Accession** | **Annotation** |
| --- | --- | --- | --- | --- | --- |
| TraesCS3D01G467600 | chr3D | 570379588 | 570380814 | tr\|M8BDU1\|M8BDU1_AEGTA | Basic 7S globulin |
| TraesCS3D01G467700 | chr3D | 570393685 | 570394941 | tr\|R7W0U4\|R7W0U4_AEGTA | Basic 7S globulin 2 |
| TraesCS3D01G467800 | chr3D | 570399910 | 570401342 | tr\|M7ZV29\|M7ZV29_TRIUA | Basic 7S globulin 2 |
| TraesCS3D01G467900 | chr3D | 570495162 | 570496385 | tr\|R7W0U4\|R7W0U4_AEGTA | Basic 7S globulin 2 |
| TraesCS3D01G468000 | chr3D | 570530479 | 570534570 | tr\|G7J516\|G7J516_MEDTR | 4-coumarate:CoA ligase-like protein |
| TraesCS3D01G468100 | chr3D | 570559188 | 570560459 | tr\|R7W0U4\|R7W0U4_AEGTA | Basic 7S globulin 2 |
| TraesCS3D01G468200 | chr3D | 570570546 | 570571577 | tr\|N1R545\|N1R545_AEGTA | Basic 7S globulin 2 |
| TraesCS3D01G468300 | chr3D | 570772456 | 570773917 | tr\|N1R545\|N1R545_AEGTA | Basic 7S globulin 2 |
| TraesCS3D01G468400 | chr3D | 570801243 | 570803210 | tr\|A0A072URN4\|A0A072URN4_MEDTR | Myb transcription factor |
| TraesCS3D01G468500 | chr3D | 570803862 | 570806999 | tr\|M8CI89\|M8CI89_AEGTA | Transcription initiation factor TFIID subunit 9 |
| TraesCS3D01G468600 | chr3D | 570839712 | 570840122 | tr\|B6TYC1\|B6TYC1_MAIZE | Oral cancer overexpressed protein 1 |
| TraesCS3D01G468700 | chr3D | 571014565 | 571014936 | AT1G34540.1 | cytochrome P450, family 94, subfamily D, polypeptide 1 |
| TraesCS3D01G468800 | chr3D | 571130776 | 571139282 | tr\|Q9LG50\|Q9LG50_ORYSJ | NBS-LRR disease resistance protein-like |
| TraesCS3D01G468900 | chr3D | 571208023 | 571209066 | AT2G05970.1 | F-box protein (DUF295) |
| TraesCS3D01G469000 | chr3D | 571211796 | 571212578 | sp\|A4XWM0\|NAPA_PSEMY | Periplasmic nitrate reductase |
| TraesCS3D01G469100 | chr3D | 571235770 | 571240717 | tr\|C6FF77\|C6FF77_SOYBN | NBS-LRR disease resistance protein |
| TraesCS3D01G469200 | chr3D | 571361412 | 571377048 | AT5G38350.1 | Disease resistance protein (NBS-LRR class) family |
| TraesCS3D01G469300 | chr3D | 571493837 | 571504029 | tr\|Q9LG50\|Q9LG50_ORYSJ | NBS-LRR disease resistance protein-like |
| TraesCS3D01G469400 | chr3D | 571514195 | 571514689 | tr\|G7IWQ8\|G7IWQ8_MEDTR | F-box protein |
| TraesCS3D01G469500 | chr3D | 571556783 | 571568177 | AT5G38350.1 | Disease resistance protein (NBS-LRR class) family |
| TraesCS3D01G469600 | chr3D | 571592337 | 571592723 | tr\|A0A0A9BFA7\|A0A0A9BFA7_ARUDO | Rpl2 |
| TraesCS3D01G469700 | chr3D | 571592359 | 571592856 | tr\|K7UGJ4\|K7UGJ4_MAIZE | 50S ribosomal protein L2 |
| TraesCS3D01G469800 | chr3D | 571592873 | 571593484 | tr\|F2WRT4\|F2WRT4_9POAL | 30S ribosomal protein S19, chloroplastic |
| TraesCS3D01G469900 | chr3D | 571678281 | 571678748 | tr\|G7IWQ8\|G7IWQ8_MEDTR | F-box protein |
| TraesCS3D01G470000 | chr3D | 571822230 | 571822932 | tr\|B9I9X0\|B9I9X0_POPTR | Hydroxyproline-rich glycoprotein |
| TraesCS3D01G470100 | chr3D | 571991747 | 571995686 | tr\|A0A080YUV7\|A0A080YUV7_WHEAT | Calcium-transporting ATPase |
| TraesCS3D01G470200 | chr3D | 572044904 | 572049382 | tr\|B9S337\|B9S337_RICCO | Sugar transporter, putative |
| TraesCS3D01G470300 | chr3D | 572100682 | 572103002 | tr\|B9S337\|B9S337_RICCO | Sugar transporter, putative |
| TraesCS3D01G470400 | chr3D | 572425625 | 572426051 | sp\|Q3MUY2\|PIGY_HUMAN | Phosphatidylinositol N-acetylglucosaminyltransferase subunit Y |
| TraesCS3D01G470500 | chr3D | 572487176 | 572490114 | tr\|M7ZW96\|M7ZW96_TRIUA | Nascent polypeptide-associated complex subunit alpha-like protein |
| TraesCS3D01G470600 | chr3D | 572507731 | 572510389 | tr\|M8AXC6\|M8AXC6_TRIUA | Nascent polypeptide-associated complex subunit alpha-like protein |

The region was determined by RAC875_c3187_873 and CAP8_c7053_252, two monomorphic markers that encompass the core 3DL QTL region.

**Fig. S1** QTL profiles for FHB traits on the 3BL and 3DL QTL region. Genetic distances are shown in centimorgans to the left of the chromosomes. A threshold of 3.0 is indicated by a dashed vertical line in the LOD graphs. Letters in parenthesis following marker names indicate the resistant (R), susceptible (S) or unknown (U) genotype of Frontana at the corresponding locus.

**Fig. S2** Physical positions of markers in the 3DL QTL region in Chinese Spring IWGSC RefSeq v.1.0 and *Aegilops tauschii*. Physical positions are denoted in the *outer* sides and marker names *inner* sides. Polymorphic markers in the ‘NASMA’ × ‘IAS20*5/H567.71’ population are *bolded* and highlighted in *red*, whereas monomorphic markers are not.

**Fig. S3** Comparison of reported QTL related to FHB traits on chromosomes 3BL and 3DL, with their physical positions projected to the IWGSC RefSeq v1.0. Molecular markers are indicated to the *right* of the chromosomes whereas their physical positions are shown to the *left*. Markers in the QTL regions reported in the current study are *bolded* and highlighted in *red*, and the position of centromere is indicated with an *oval circle*. For each QTL, the corresponding resistance source, type of resistance and citation are denoted.

[1] Buerstmayr M, Buerstmayr H (2015) Comparative mapping of quantitative trait loci for Fusarium head blight resistance and anther retention in the winter wheat population Capo x Arina. Theor Appl Genet 128:1519-1530

[2] Cai J, Bai G (2014) Quantitative trait loci for fusarium head blight resistance in Huangcandou × ‘Jagger’ wheat population. Crop Sci 54:2520-2528

[3] Islam MS, Brown-Guedira G, van Sanford D, Ohm H, Dong Y, McKendry AL (2016) Novel QTL associated with the Fusarium head blight resistance in Truman soft red winter wheat. Euphytica 207:571-592

[4] Li X, Xiang ZP, Chen WQ, Huang QL, Liu TG, Li Q, Zhong SF, Zhang M, Guo JW, Lei L, Luo PG (2017) Reevaluation of two quantitative trait loci for type II resistance to fusarium head blight in wheat germplasm PI 672538. Phytopathology 107:92-99

[5] Liu S, Griffey CA, Hall MD, McKendry AL, Chen J, Brooks WS, Brown-Guedira G, Van Sanford D, Schmale DG (2013) Molecular characterization of field resistance to Fusarium head blight in two US soft red winter wheat cultivars. Theor Appl Genet 126:2485-2498

[6] Paillard S, Schnurbusch T, Tiwari R, Messmer M, Winzeler M, Keller B, Schachermayr G (2004) QTL analysis of resistance to Fusarium head blight in Swiss winter wheat (Triticum aestivum L.). Theor Appl Genet 109, no 2:323-332

[7] Petersen S, Lyerly JH, McKendry AL, Islam MS, Brown-Guedira G, Cowger C, Dong Y, Murphy JP (2017) Validation of Fusarium head blight resistance QTL in US winter wheat. Crop Sci 57:1-12

[8] Somers DJ, Fedak G, Savard M (2003) Molecular mapping of novel genes controlling Fusarium head blight resistance and deoxynivalenol accumulation in spring wheat. Genome 46:555-564

[9] Yu JB, Bai GH, Zhou WC, Dong YH, Kolb FL (2008) Quantitative trait loci for Fusarium head blight resistance in a recombinant inbred population of Wangshuibai/Wheaton. Phytopathology 98:87-94

[10] Zhang X, Pan H, Bai G (2012) Quantitative trait loci responsible for Fusarium head blight resistance in Chinese landrace Baishanyuehuang. Theor Appl Genet 125:495-502

[11] Kollers S, Rodemann B, Ling J, Korzun V, Ebmeyer E, Argillier O, Hinze M, Plieske J, Kulosa D, Ganal MW, Roder MS (2013) Whole genome association mapping of Fusarium head blight resistance in European winter wheat (Triticum aestivum L.). Plos One 8:e57500

[12] Shen X, Ittu M, Ohm HW (2003) Quantitative trait loci conditioning resistance to Fusarium head blight in wheat line F201R. Crop Sci 43:850-857

[13] Jia G, Chen PD, Qin GJ, Bai GH, Wang X, Wang SL, Zhou B, Zhang SH, Liu DJ (2005) QTLs for Fusarium head blight response in a wheat DH population of Wangshuibai/Alondra's'. Euphytica 146:183-191

[14] Draeger R, Gosman N, Steed A, Chandler E, Thomsett M, Srinivasachary, Schondelmaier J, Buerstmayr H, Lemmens M, Schmolke M, Mesterhazy A, Nicholson P (2007) Identification of QTLs for resistance to Fusarium head blight, DON accumulation and associated traits in the winter wheat variety Arina. Theor Appl Genet 115:617-625

[15] Klahr A, Zimmermann G, Wenzel G, Mohler V (2007) Effects of environment, disease progress, plant height and heading date on the detection of QTLs for resistance to Fusarium head blight in an European winter wheat cross. Euphytica 154:17-28

[16] Holzapfel J, Voss HH, Miedaner T, Korzun V, Haberle J, Schweizer G, Mohler V, Zimmermann G, Hartl L (2008) Inheritance of resistance to Fusarium head blight in three European winter wheat populations. Theor Appl Genet 117:1119-1128

[17] Szabo-Hever A, Lehoczki-Krsjak S, Toth B, Purnhauser L, Buerstmayr H, Steiner B, Mesterhazy A (2012) Identification and validation of fusarium head blight and Fusarium-damaged kernel QTL in a Frontana/Remus DH mapping population. Can J Plant Pathol 34:224-238

[18] Lu Q, Lillemo M, Skinnes H, He X, Shi J, Ji F, Dong Y, Bjornstad A (2013) Anther extrusion and plant height are associated with Type I resistance to Fusarium head blight in bread wheat line 'Shanghai-3/Catbird'. Theor Appl Genet 126:317-334

[19] Li T, Zhang DD, Zhou XL, Bai GH, Li L, Gu SL (2016) Fusarium head blight resistance loci in a stratified population of wheat landraces and varieties. Euphytica 207:551-561
